# Structure-Guided In-Use Stability Assessment of Monoclonal Antibody Tislelizumab

**DOI:** 10.3390/ph18101539

**Published:** 2025-10-13

**Authors:** David Andre Rudd, Ghizal Siddiqui

**Affiliations:** 1 Drug Delivery, Disposition and Dynamics, Monash Institute of Pharmaceutical Sciences, Monash University, Parkville, VIC 3052, Australia; ghizal.siddiqui@monash.edu; 2 Medicines Manufacturing Innovation Centre, Australian Synchrotron, Clayton, VIC 3168, Australia; 3 Drug Target Identification Platform, Monash Institute of Pharmaceutical Sciences, Monash University, Parkville, VIC 3052, Australia

**Keywords:** monoclonal antibody, structural stability, tislelizumab, biologic, immunotherapy

## Abstract

**Background/Objectives**: Monoclonal antibody (mAb) stability is critical not only during manufacturing but also at the point of clinical administration. For therapies like tislelizumab (Tevimbra), a programmed death-1 (PD-1) targeting IgG mAb, delays in dosing often result in prepared infusions being discarded, contributing to substantial drug waste despite being engineered for improved stability. **Methods**: To evaluate the physicochemical in-use stability of tislelizumab in a ready-to-administer format, we mapped degradation pathways, including post-translational modifications (PTMs); peptide alterations; pH and solution characteristics—under 12-month storage (ultra-long), under 1-month storage (0, 7, 14, 21, 28 and 31 days), and under exposure-related forced degradation conditions including room temperature, elevated temperature, pH (acidic/basic), oxidation and UV exposure. Structural analysis was contextualised to the known PD-1 binding site, making stability assessment relevant to tislelizumab’s mechanism-of-action in blocking PD-1. To assess solution stability, a validated size-exclusion chromatography (SEC) assay was applied to all conditions. **Results**: Aggregation was identified as the primary degradation pathway during ultra-long-term storage. SEC and chemical assessment revealed no measurable changes in protein quantity, aggregation, peptide integrity, or PTM profile over 31 days at 2–8 °C in polyolefin intravenous bags (1.6 mg/mL). **Conclusions**: These results support the structural and physicochemical stability of tislelizumab under refrigerated conditions.

## 1. Introduction

Cancers, as a disease group, are collectively one of the leading causes of death globally [1], with an increasing incidence and mortality. As the health burden of cancers has increased, there has been an increasing cost in cancer treatment, with cancer care expected to have an estimated economic expenditure of $25.2 trillion (international dollars; 2020–2050) [1]. Part of the cost associated with cancer care is the increasing price of approved, yet efficacious, therapies that have a high manufacturing cost, including immunotherapies [2]. While better treatment options are part of the solution for cancer care, minimising any unnecessary costs in their production and clinical use, where safe to do so, should be explored in this context. One particular aspect of the preparation of biologics for treatment is clinical dosing practices, where there is often minimal flexibility in how a therapy is formulated and administered to a patient despite new engineering approaches that improve intrinsic stability. Once a dose is made up for IV infusion, patient criteria may delay administration, yet formulated products have a short-designated expiry and must be discarded if not administered, with a default expiry of ≤24 h at 2–8 °C.

There have been numerous initiatives to address the cost of immunotherapies in clinical use. This has included (i) adaptive and fixed dosing, where changes to dose/weight have allowed more controlled management of medications [3,4]; (ii) dose-rounding, where doses are rounded to the nearest vial size [5]; (iii) extension of expiry after re-evaluation of stability (reported through therapeutic regulatory agencies); and (iv) extension of formulation expiry made up for IV administration [6]. It has been suggested that the latter initiative, extension of in-use products, must address multiple factors related to the clinical setting, including the stability of the product as an in-use solution [6]. Previous studies have found that ‘beyond-use dating’ should be governed by the shortest of the dates attributed to each of these factors. The analytical techniques used to determine stability should include measurements of concentrations that include determining aggregation effects; for example, size-exclusion chromatography coupled to ultraviolet (UV)-visible absorption spectroscopy that can measure changes in monomer, dimer, and higher/lower-molecular-weight species (H/LMWS). In addition to concentration, other critical quality attributes (CQAs) that measure changes to the mAbs, that can influence dose, efficacy, and immunogenicity, should be considered. Studies have shown that in-use solutions of mAbs can remain physically and chemically stable for extended periods of time under refrigeration, including infliximab biosimilar [7] (1 month), bevacizumab biosimilar [8] (6 weeks), trastuzumab biosimilar [9,10] (77 and 28 days), nivolumab [11] (7 days—PD-1 antibody), and trastuzumab [12] (6 months) as examples. A recent review by Le Basle et al. [13], has suggested that most of the reviewed antibodies studied are a lot more stable once diluted than what is included on the product characteristics summary, particularly with newer engineered biologics that substitute amino acids prone to oxidation/deamidation (i.e., methionine). Where stability is extended in clinical settings, it is essential that diluted solutions are prepared aseptically and stored to prevent microbial risk.

Tislelizumab is a monoclonal immunoglobulin G antibody that targets the programmed death receptor-1 (PD-1), blocking interaction with the programmed death ligand-1 (PD-L1; also known as CD274 and B7-H1) [14]. PD-1 is expressed on T-cells, while PD-L1 is expressed in tissues and can be upregulated on cancer cells (PD-L1 positive), where interaction between the two inhibits T-cell activation [14] and thus inhibits antitumour immunity. Immune checkpoint-inhibiting antibodies are now established US Food and Drug Administration (FDA)-approved cancer immunotherapies that have been found to be beneficial in both PD-L1 positive and PD-L1 negative patients [15]. The structure of tislelizumab has been designed to inhibit the binding of PD-1 to PD-L1, while reducing binding interactions with Fc-gamma receptors (FcγR). The design structure of tislelizumab prevents the downregulation of T-cell responses that allow cancerous cells to evade the immune system, while also preventing the rapid removal of administered tislelizumab from the body via a pathogen recognition pathway [16].

The specific structural design of tislelizumab is an essential part of its efficacy in this dual role and could be considered a critical aspect of its use as a chemotherapeutic drug. To date, tislelizumab has been approved in China for the treatment of squamous/non-squamous non-small-cell lung cancer, gastroesophageal adenocarcinoma, classical Hodgkin’s lymphoma, urothelial carcinoma, hepatocellular carcinoma, and microsatellite instability-high/mismatch repair-deficient solid tumours by the National Medical Product Administration (NMPA) [16,17]. Additionally, the US Food and Drug Administration (FDA) has granted tislelizumab orphan designation for the treatment of oesophageal cancer and gastric cancer, including cancer of the gastroesophageal junction [16]. In the majority of the clinical trials mentioned above, tislelizumab is administered as an IV infusion of 200 mg at predefined dosing points.

For tislelizumab (Tevimbra^®^), the current label specifies that diluted infusions should be used within 24 h when stored at 2–8 °C, consistent with other PD-1 antibodies such as nivolumab and pembrolizumab. These short in-use constraints are applied conservatively to mitigate safety risks but often underestimate the true molecular stability of engineered antibodies. As a result, prepared tislelizumab are discarded in clinical settings if not delivered to patients in a timely manner (transport from clinical site to patient) or if dosing is delayed or rescheduled, despite growing evidence that PD-1 antibodies maintain stability well beyond these limits under controlled conditions.

In this study, we assess the physicochemical stability of tislelizumab, ready for infusion, in the context of maintaining the efficacy of tislelizumab in binding to PD-1 using advanced analytical techniques not routinely used in stability assessment of biologics, including PTM mapping and ion mobility spectroscopy. For storage stability, this study developed a validated (ICH-Q2, R1) SEC assay to assess tislelizumab across a one-month period (31 days), when stored in 2–8 °C, within polyolefin bags at a nominal concentration of 1.6 mg/mL (2× tislelizumab 10 mg/mL vials (200 mg) in 100 mL IV freeflex^®^ bags (125 mL with vial volume and overage)). To identify aggregation as a suitable measure of tislelizumab stability, comparative samples (*n* = 4) were made up and stored for 12 months for comparison to a 31-day storage period. In addition to ultra-long-storage, exposure-related forced degradation conditions were applied to infusion-ready tislelizumab including acidic (HCl, low/high), basic (NaOH, low/high), accelerated temperature (25 °C ± 2 °C; 40 °C ± 2 °C; 50 °C ± 2 °C), oxidation (H_2_O_2_, low/high), and UV (photostability as described in ICH Q1B; 1.2 million lux·h + 200 W·h/m^2^ UV) to establish assays as stability-indicating. Peptide mapping, with a combination of data-independent and data-dependent acquisition (DIA/DDA), was applied to forced degradation and storage conditions to generate a PTMs map for tislelizumab. Changes to PTMs for all conditions were specifically mapped to amino acids involved in PD-1 binding and critical amino acids involved in maintaining the higher-order structure of antibodies more generally. Other general physical characteristics were measured for an overall description of stability including turbidimetry, pH, particle formation (dynamic light scattering (DLS)), and higher-order structure (nano differential scanning fluorimetry; nanoDSF).

## 2. Results

Forced exposure degradation and ultra-long storage (12 months) at the recommended temperature (2–8 °C) showed that peptide mapping and SEC is an effective combination for readily identifying changes in tislelizumab stability.

### 2.1. Forced Degradation

**Figure 1:**
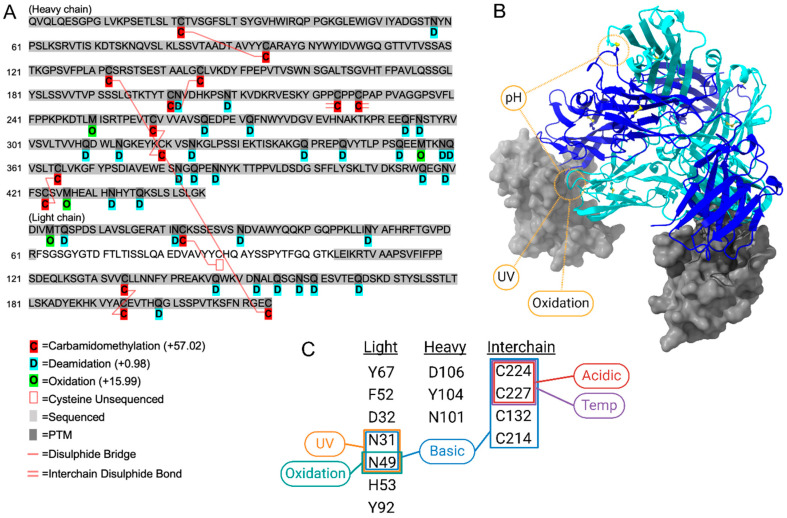
Tislelizumab sequence (KEGG Entry D11487), (**A**), with a post-translational modification (PTM) map generated using a combination of data-independent and data-dependant acquisition from forced exposure degradation and ultra-long storage as an in-use solution. Structure of tislelizumab Fab bound to PD-1 (PDB: 7CGW), (**B**), showing crucial interaction sites between the light chain (cyan ribbon) and heavy chain (blue ribbon) of tislelizumab and PD-1 (grey surface rendered). Changes in pH, UV, and oxidation (deamination as a secondary effect) are degradation factors that would most affect tislelizumab’s mechanism-of-action. Key amino acids that affect binding and the higher-order structure of tislelizumab are listed and matched against all identified PTMs, (**C**), PTM assignment available in the Supplementary Materials.

Peptide mapping (Figure 1) with close monitoring of PTMs in the light chain from forced degradation samples can identify when amino acids responsible for PD-1 binding are affected by instability, including asparagine 31 and 49 which are both involved in hydrogen bonding with PD-1 [17]. Conditions including UV exposure, oxidation (via a secondary effect) and a change in pH to more basic conditions can specifically affect these two amino acids (Figure 1B,C), indicating that in-use infusions should be protected from both chemical- and UV-induced oxidation and long periods of elevated temperature, which also increased pH values. While asparagine is not directly oxidised, in protein therapeutics and stability studies, oxidative stress testing (e.g., H_2_O_2_ treatment) can reveal not just methionine oxidation but also enhanced asparagine deamidation as a secondary effect.

**Figure 2:**
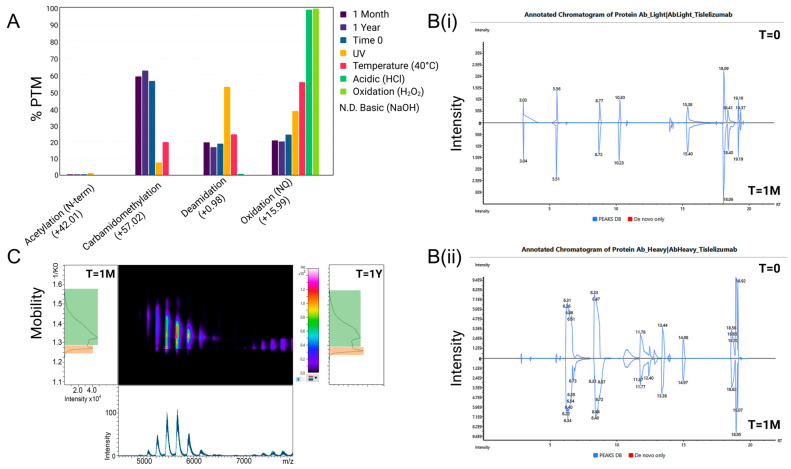
Effect of time, temperature, UV, oxidation, and pH on the intrinsic stability of tislelizumab as an in-use solution. The proportion of PTM from pooled stability samples, (**A**), shows the impacts of time and chemical condition on tislelizumab (Supplementary Materials). Representative peptide chromatograms show matching sequences for time zero and one month in optimal storage for the light chain, (**B(i)**), and the heavy chain, (**B(ii)**). Ion mobility-MS analysis of intact tislelizumab, (**C**), shows aggregation to be the main stability-indicating effect for storage over time, where a one-year sample has an increase in the aggregate mobility area relative to the monomer compared to one month.

More general impacts of exposure-related degradation also target interchain disulphide bridges, including pH changes and accelerated temperature. It is these effects that impact aggregation properties and lead to a gradual shift towards particle formation. Of all the degradation parameters assessed, UV had the greatest impact on increasing deamidation and N-terminal acetylation, while temperature, acidic conditions, and chemical oxidation increased oxidation on multiple asparagine and glutamine residues across both the light and heavy chains of tislelizumab (Figure 1 and Figure 2). Strong basic conditions led to a complete precipitation of tislelizumab, producing an opaque solution with few matching detectable peptides. Under optimal storage conditions, one month showed minimal changes in the proportion of PTM when compared to time zero, while one year of storage showed a very slight increase in the proportion of carbamidomethylation (Figure 2), which can be indicative of disulphide bond instability and degradation of the tertiary structure when sample preparation conditions are kept consistent across treatment and control samples. A shift towards aggregation during ultra-long storage as an in-use solution was also identified using TIMS-MS analysis, where an increase in multiply-charged clusters during ion mobility separation was found at 1.25–1.28 1/K0 mobility when compared to time zero and one month (Figure 2C).

### 2.2. Quantitative Stability Assessment

**Figure 3:**
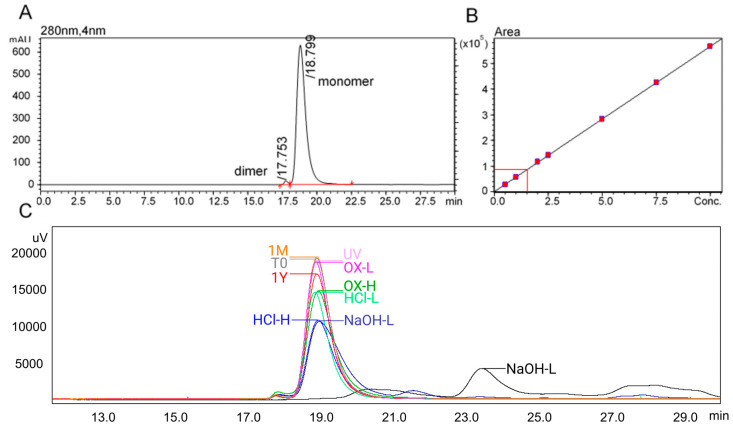
SEC-UV chromatogram of tislelizumab (**A**) at time zero showing 99.05% monomer (18.80 min) and 0.95% dimer (17.75 min) content. (**B**) Tislelizumab was quantifiable between 0.5 and 10 mg/mL (r = 0.99999; 0.86%RSD), with a time zero concentration of 1.535 mg/mL (95.94% recovery from a nominal 1.6 mg/mL – including transfer into IV bags). Loss of monomer content, from both time and chemical degradation is shown in (**C**).

Monomer concentration was quantified using a validated SEC-UV method (r = 0.99999, RSD = 0.86%). At time zero, 99.05% of tislelizumab existed in the monomeric form, with 0.95% present as dimer, Figure 3. Across a 31-day refrigerated stability study, no significant differences were found in monomer content, protein concentration, or aggregation (one-way ANOVA, *p* = 0.62; regression analysis, slope not significantly different from zero, *p* = 0.41). For the comparison between day 0 and day 31, the mean difference in monomer content was –0.01% (95% CI: –0.08% to +0.06%, unpaired *t*-test, *p* > 0.05). These results confirm the absence of a statistically meaningful change in protein quantity or aggregation over the test period. The formulation remained clear, colourless, and free of visible particles, with consistent pH (6.37), low particle burden (DLS) [18], and unchanged unfolding temperatures (nanoDSF), as shown in Table 1.

Chemical and structural integrity were retained across all measured CQA under optimal 2–8 °C storage, Table 1. At RT conditions, even short periods are not recommended, as 24 h at room temperature led to an elevation in average particle size (~20% increase, limit of acceptance criteria), pH (+0.18), and Tm^2^ unfolding temperature (+2.4 °C), indicating CQA are affected by short periods of heat that would be unrecoverable. Stability under optimal conditions for one month contrasts sharply with the accelerated and forced degradation conditions, where chemical stressors (base, acid, oxidants) and physical exposure (UV, heat) caused losses in monomer and peptide signals. These data collectively demonstrate that aggregation, pH, particle size, and PTM modifications are stability-indicating effects and that the selected analytical platform is suitable for detecting degradation in tislelizumab. However, longer stability beyond 31 days is uncertain, as ultra-long storage for one year did not maintain concentration, although still within the conventional acceptance criteria for loss of content [19]. Although the 12-month samples technically remained within certain acceptance criteria, the observed shifts in aggregation and chemical modification indicate that a one-year extension of beyond-use dating would not be justifiable in a clinical setting or from a regulatory point of view. Instead, our findings should be interpreted as confirming that the selected assays are stability-indicating, rather than evidence to support safe clinical use beyond 31 days.

## 3. Discussion

The stability of immunotherapies plays a critical role in ensuring their therapeutic efficacy. Modern engineered mAb have been specifically designed to improve stability in addition to extending pharmacokinetic profiles, where conventional stability parameters and expiries should be reconsidered with this in mind. Replacement of vulnerable methionine on engineered IgG improves the potential for oxidation at these sites, thereby improving stability of CQA. As such, any changes in manufacturing, formulation, or storage, including in-clinic preparation and storage of infusion-ready solutions, should be open for re-evaluation. Intrinsic stability should have a strong focus on the attributes involved in its mechanism-of-action. In the case of tislelizumab, it is the PD-1 binding site. Significant effort goes into developing mAb formulations with extended shelf lives under controlled conditions (typically 2–8 °C), particularly given the high cost of manufacturing these biologics. However, it is inconsistent to maintain such stable formulations only to implement very short in-use expiry durations post-preparation, especially when the three major risk factors; (i) intrinsic stability; (ii) aseptic preparation; and (iii) patient safety, can be mitigated.

In this study, tislelizumab demonstrated no measurable changes in concentration, physicochemical parameters (e.g., pH), or CQAs related to structure and aggregation during one month of refrigerated storage at infusion concentration. In contrast, samples subjected to accelerated conditions (room temperature), forced degradation (chemical stress), and ultra-long storage exhibited unacceptable changes in aggregation and structural integrity, as expected. Exceeding these predefined CQA thresholds is clinically relevant, as loss of monomer content or increased aggregation not only reduces the effective delivered dose but also elevates the risk of immunogenicity, both of which may compromise the therapeutic efficacy and safety profile of tislelizumab.

A limitation of this study is the absence of a functional bioassay, and future work should include PD-1 binding or cell-based PD-1/PD-L1 inhibition assays to confirm that structural stability is predictive of retained bioactivity.

## 4. Materials and Methods

Tislelizumab was provided by BeiGene as a 10 mg/mL solution for IV infusion (Batch 24848.5). Samples were made using sterile syringes and needles (3 mL; 23 G, Terumo, Macquarie Park, Australia) and diluted into polyolefin IV bags containing 0.9% *w*/*v* NaCl (2 × 10 mg/mL vials/100 mL + overage (~120 mL); freeflex^®^) under aseptic conditions. Refrigerated storage (2–8 °C) was achieved with a scientific refrigerator (TR-240-IS; Thermoline, Macquarie Park, Australia) with temperature monitoring (Clever Logger; Castle Hill, Australia). Room-temperature samples were maintained in stability cabinets at 25 °C (±2 °C), and heat-stressed samples were incubated in a Thermo Scientific heating oven. A USP monoclonal IgG (system suitability—1445550) was used as a system suitability and reference standard. The 0.9% *w*/*v* sodium chloride from IV bags was used as a diluent.

Stability and forced degradation samples: Forced degradation samples were made according to the conditions recommended in the stability review by le Basle et al. [13], including acidic (HCl—low 0.01 M, high 0.1 M); basic (NaOH—low 0.01 M, high 0.1 M); accelerated temperature (25 °C ± 2 °C; 40 °C ± 2 °C; 50 °C ± 2 °C); oxidation (H_2_O_2_—low, 0.1%, high, 1%), and UV (photostability as described in ICH Q1B; 1.2 million lux·h + 200 W·h/m^2^ UV) conditions. Forced degradation conditions were applied for 24 h, with the exception of room-temperature stress which included a 7- and 14-day interval in addition to 24 h. Stability at the recommended 2–8 °C was conducted at 0, 24 h and 7, 14, 21, 28 and 31 days post formulation. Ultra-long stability, as a way of determining if selected assays were stability-indicating when tislelizumab is stored for longer than recommended, was conducted at 12 months.

Size-exclusion chromatography: SEC mobile phase was made using ultrapure water from a Milli-Q system fitted with an LC-Pak^®^ polisher (Merck Millipore; Burlington, MA, USA). Sodium dodecyl sulphate (≥99%, dust free pellets; 100 mM) and sodium phosphate monobasic (≥99%, BioXtra, Seneffe, Belgium; 100 mM) were obtained from Sigma-Aldrich, Bayswater, Australia. Mobile phases were filtered with PTFE membrane filters (Omnipore™, Newnan, GA, USA, 47 mm; Merck, Macquarie Park, Australia) using Aldrich glass filtration apparatus (1 L) attached to a vacuum pump (Air Admiral^®^, Cole-Palmer^®^; Vernon Hills, IL, USA). Samples were prepared for injection in 2 mL certified autosampler vials with PTFE bonded polypropylene caps lined with Si septa (Shimadzu; Kyoto, Japan). SEC was achieved using a TSKgel G3000SWxl column (7.8 mm I.D. × 30 cm, 5 µm particle size; #005D02405D, Yamaguchi, Japan).

An IgG reference standard was made as a 10 mg/mL concentration using IV solution as the diluent, designated as the reference standard (RS). The RS solution was then diluted to 75, 50, 25, 20, 10 and 5% RS (RS calibration curve). Samples were run between two calibration curves including an initial RS system suitability standard (*n* = 3) before the calibration curve and before and after a batch of samples. Each treatment was replicated four times, and each replicate was run in duplicate. Inter-run quality control (QC) samples were added between every 10 samples.

Samples were run on a Shimadzu Nexera UHPLC (Shimadzu, Kyoto, Japan) with diode array detector with the following components: DGU-20A degassing unit, LC-30AD binary pumps, SIL-30AC autosampler, CBM-20A communication module, CTO-20AC column oven, SPD-M30A DAD and a 20 µL mixing chamber. Tislelizumab was monitored at 280 nm. Optimisation of flow rate, mobile phase additives, injection volumes, run time and autosampler conditions were initially tested. Chromatography was achieved using isocratic flow of a 100 mM sodium phosphate plus 100 mM sodium dodecyl sulphate solution at a flow rate of 0.3 mL/min for 30 min. The autosampler temperature was set to 15 °C and the column oven to 30 °C. The injection volume was fixed at 2 µL. Autosampler stability was conducted over 24 h. Stability timepoints included day 0, 1, 7, 14, 21, 28 and 31 days post formulation. All data were acquired, processed, and quantified using LabSolutions (Shimadzu, Kyoto, Japan) using the Quant Browser function.

Peptide mapping using DDA and DIA with PTM assignment: Tislelizumab samples were initially quantified using HPLC-UV. A total of 50 ug of samples were resuspended in 100 Mm 4-(2-Hydroxyethyl)-1-piperazineethanesulfonic acid (HEPES) and then reduced and alkylated using tris(2-carboxyethyl) phosphine (TCEP) and chloroacetamide at 10 mM and 40 mM final concentration. Following which, samples were digested overnight with sequencing grade trypsin (Promega, Alexandria, Australia). After digestion, the sample solution pH was adjusted to between 2 and 4 using formic acid (FA). In-house stage tips were generated using 2 layers of C18 membrane (CDS Empore™, Thermo Fisher Scientific, Waltham, MA, USA) in 200 μL pipette tips. The stage tips were wetted using 40 μL methanol, followed by centrifugation at 1200× *g* for 1 min. The membrane was equilibrated using 40 μL of 0.1% FA before centrifugation at 1200× *g* for 1 min. The samples were loaded before spinning at 800× *g* for 5 min. To remove excess NaCl, samples were washed four times with 40 μL of 0.1% formic acid, followed by a consecutive 1 min centrifugation at 1200× *g*. For elution, a mixture of 2 × 50 μL 70% ACN with 0.1% FA was used, each centrifuged at 500× *g* for 5 min. Extracted peptides were dried in a Speed-Vac (Labconco, Kansas City, MO, USA) and stored at −20 °C until analysis.

On the day of analysis, samples were reconstituted in 10 μL of 2% acetonitrile (ACN) with 0.1% FA. Indexed retention time (iRT; Pierce™, Thermo Fisher Scientific) peptides were added prior to LC-MS/MS analysis on an Orbitrap Astral mass spectrometer with nanoLC (Thermo Fisher Scientific). Samples were loaded at a flow rate of 15 μL/min onto an Aurora Ultimate 25 cm × 75 μm C18 UHPLC column (IonOpticks, Collingwood, Australia). Peptides were separated using a gradient of water (mobile phase A) and 80% ACN (mobile phase B). The gradient profile included the following: 0 min (4% B), 5 min (10% B), 6 min (10% B), 16.1 min (28% B), 17.6 min (42% B), 18.6 min (99% B), followed by a column wash up to 20 min (99 B) and equilibration back to starting conditions. Acquisition was performed initially in a data-independent acquisition (DIA) mode to establish sequence coverage using Spectronaut Pulsar (v16.1, Biognosys, Schlieren, Switzerland) (version 19.2.240905.62635) in Direct DIA mode and linked to the human protein database (Homo sapiens, uniprot-proteome_UP000005640.fasta). For DIA, MS1 spectra were collected in the Orbitrap at a resolving power of 240,000 over *m*/*z* 380–980. The MS1 normalised AGC target was set to 500% with a maximum injection time of 5 ms. DIA MS2 scans were acquired in the Astral analyser over a 380–980 *m*/*z* range with a normalised AGC target of 800% and a maximum injection time of 3 ms and an HCD collision energy setting of 25% and a default charge state of +2. Window placement optimisation was turned on with isolation widths of 2 Th. Samples were then run in data-dependent acquisition mode (DDA) to specifically monitor for PTMs known to be involved in degradation, including sequence regions involved in tislelizumab binding. In DDA mode, the Orbitrap Astral MS was operated in positive mode with a fixed cycle time of 0.5 s with a full scan range of 400–1500 *m*/*z* at a resolution of 240,000. The automatic gain control (AGC) was set to “custom”, with a normalised AGC target of 500% and a maximum injection time of 5 ms. Peptide fragmentation was triggered by higher-energy collisional dissociation (HCD) with an HCD collision energy set at 25%. Fragment ion scans were recorded with the Astral analyser with a scan range of 110–2000 *m*/*z*. Modified peptide sequences (and protein identity) were determined using PEAKS software (PEAKS^®^ Studio 13; Bioinformatics Solutions Inc., Waterloo, ON, Canada) and default settings by matching to the human protein database (Homo sapiens, uniprot-proteome_UP000005640.fasta) and label-free quantification of identified proteins and peptides were then performed.

pH measurement: The pH of antibody formulations was monitored at each designated stability timepoint, including initial (T = 0), accelerated temperature, long-term (2–8 °C), and for forced degradation conditions. Measurements were performed using a calibrated pH metre equipped with a low-binding, protein-compatible microelectrode (Thermo Fisher Orion™ ROSS PerpHecT™ Micro Electrode; Waltham, MA, USA). All measurements were conducted at controlled room temperature (20–25 °C) to minimise temperature-related variability. Samples were analysed in their original formulation matrices without dilution.

Particle size measurement using dynamic light scattering (DLS) and Trapped Ion Mobility Spectrometry-Mass Spectrometry (TIMS-MS): Particle size characteristics were measured using a Malvern Zetasizer (Nano-ZS; ATA Scientific, Caringbah, Australia). The measurement SOP was based on protein analysis in a NaCl dispersant solution. Dispersant settings included the following: 45 °C, 0.891 cP viscosity and a refractive index of 1.33. Measurements were conducted in disposable low volume cuvettes (ZEN0040) with a 173° backscatter. Each sample was run twelve times, with a 10 s/run period. Attenuation was selected automatically, and data were acquired in multiple narrow modes (high-resolution). A reference standard was used to determine optimal settings showing a size range between 7.5 and 11.7 d.nm (typically ~10 d.nm [17]) in IV solution.

To evaluate aggregation phases leading to particle formation (general charge state distribution), tislelizumab from each condition was buffer exchanged into 150 mM ammonium acetate for direct infusion into a timsTOF Flex MALDI-2 (Bruker; Billerica, MA, USA). Instrumental conditions were optimised from default mAb ESI-TIMS-MS methods including the following: spray voltage, 4.0 kV; capillary temperature, 150 °C; nebuliser gas pressure, 0.4 bar; drying gas flow, 5 L/min; TIMS voltage ramp settings, start 200 V, end 1800 V; TIMS accumulation time, 200 ms; m/z range, 900–8000; positive ion mode; TIMS separation, 0.8–1.5 V·s/cm^2^. Spectra were resolved by averaging in Data Analysis (Bruker).

Nano differential scanning fluorimetry (nanoDSF): Samples were taken at time 0 and 4 weeks during the stability period to measure changes in the unfolding properties of tislelizumab. Samples were loaded into Tycho NT.6 capillaries and run through a predefined gradient of 1 °C/min from 35 °C to 95 °C following the manufacturer’s recommendations for antibody characterisation.

Acceptance Criteria (Table 1): Critical quality attributes (CQAs) were based on established regulatory guidance for biotechnological products, including ICH Q5C (Stability Testing of Biotechnological/Biological Products) and EMA/FDA expectations, which recommend evaluation against predefined limits for content, purity, and structural integrity. Specifically, ±10% for protein concentration, ≥95% monomer content, ≤2% aggregation, and ≤1% fragments are commonly applied thresholds in biopharmaceutical stability studies to ensure maintenance of product quality within clinically acceptable ranges.

## 5. Conclusions

Regulatory guidance (e.g., EMA, ICH Q1E) supports a data-driven, decision-tree approach for defining product shelf life, evaluating stability across individual CQAs. Applying this framework to tislelizumab, SEC-UV analysis showed stable monomer content across all refrigerated timepoints. For accelerated temperature, minor deviations observed at room temperature remained within acceptable ICH stability thresholds; however, changes in pH under RT conditions, along with supporting data from the PTM profile, peptide composition, DLS and nanoDSF, would trigger re-evaluation and potentially disqualify RT storage. Tislelizumab appears intrinsically stable for one month, but only under optimal conditions, where exposure events like short periods of room temperature or UV exposure should be avoided. The extended stability must also balance intrinsic physicochemical stability with patient safety and aseptic handling considerations for this duration (ICH Q5C, EMA/FDA guidance).

## Figures and Tables

**Table 1:** Stability profile of tislelizumab (0–31 days, 2–8 °C) in comparison to exposure-related forces degradation samples.

	Conc.	Monomer %	Aggregate %	Fragment %	pH	Visible Appearance	Particles ^#^ (Subvisible)	Unfolding
Method	HPLC-UV	SEC	SEC	SEC	USP <791>	USP <790>	DLS	nanoDSF (Tm ^1^/Tm ^2^/Tm ^3^) °C
Acceptance criteria	±10% *	≥95%	≤2%	≤1%	±0.3	C/CL/NP +/+/+		≤3 °C Tm ^*n*^
Condition								
RS ^1^	9.997			0	6.50	+/+/+		
Time 0 ^2^	1.535 (0.28)	99.02	0.98	0	6.37	+/+/+	11.38 (2.46), 0.12	68.3/76.9/91.4
1 Day ^2^	1.539 (0.34)	99.08	0.92	0	6.37	+/+/+		
7 Days ^2^	1.535 (0.25)	99.11	0.89	0	6.37	+/+/+		
14 Days ^2^	1.543 (0.52)	99.09	0.91	0	6.37	+/+/+		
21 Days ^2^	1.548 (1.41)	99.07	0.93	0	6.37	+/+/+		
28 Days ^2^	1.536 (0.36)	99.07	0.93	0	6.37	+/+/+		
31 Days ^2^	1.523 (0.36)	99.04	0.96	0	6.37	+/+/+	12.02 (1.60), 0.15	68.6/76.8/91.4
1 Year ^2^	1.420 (2.02)	99.09	0.91	0	6.27	+/+/+	13.46 (3.67), 0.21	
RT ^3^	1.564 (0.28)	99.06	0.94	0	6.55	+/+/+	13.74 (13.58), 0.18	68.8/79.3/91.4
RT 7 Days ^3^	1.560 (0.30)	99.04	0.96	0	6.57	+/+/+		
RT 14 Days ^3^	1.568 (0.08)	99.07	0.93	0	6.59	+/+/+		
40 °C ^4^	1.761 (4.67)	94.00	6.00	0	6.45	+/+/+	57.07 (43.76), 0.12	68.8/79.0/91.5
50 °C ^4^	1.927 (7.80)	85.99	14.0	0	6.55	−/+/−	102.98 (62.23), 0.29	
Acidic Low ^4^	1.386 (0.38)	97.48	2.52	0	6.04	+/+/+	17.88 (5.40), 0.25	
Acidic High ^4^	0.938 (1.22)	98.93	1.07	0	4.98	−/+/−		
Basic Low ^4^	1.148 (0.54)	89.98	1.72	8.3	7.72	−/+/−	26.88 (43.60), 0.21	
Basic High ^4^	ND	ND	ND	100	8.70	−/+/−		
Ox Low ^4^	1.527 (0.16)	99.01	0.99	0	6.37	+/+/+	42.24 (58.65), 0.17	
Ox High ^4^	1.177 (0.39)	99.10	0.90	0	6.24	+/+/+		
UV ^4^	1.576 (0.13)	98.86	1.14	0	6.27	+/+/+	12.47 (0.84), 0.15	

* Values are mg/mL, mean stability *n* = 4 (%RSD), mean exposure degradation *n* = 3 (%RSD), nominal 1.6 mg/mL; C—clear, CL—colourless, NP—no visible particles, where (+) indicates pass, (−) indicates fail; ^#^ values are mean Z-average (%RSD), polydispersity index (PDI); ^1^ undiluted tislelizumab, 10 mg/mL; ^2^ optimal 2–8 °C (±2 °C); ^3^ room temperature—25 °C (±2 °C); ^4^ 24 h duration. ND—not detected. Acceptance criteria were defined according to ICH Q5C and commonly applied stability thresholds. Data available in the Supplementary Materials.

## Data Availability

The authors declare that all the data supporting the findings of this study are available within the paper and its Supplementary Materials.

## Supplementary Materials

The following supporting information can be downloaded at https://www.mdpi.com/article/10.3390/ph18101539/s1, File S1: Data outputs for DIA and DDA sequencing data for PTM assignment, timsTOF intact analysis, DLS, and nanoDSF.
